# Characterisation of *Bordetella bronchiseptica* isolated from rabbits in Fujian, China

**DOI:** 10.1017/S0950268820001879

**Published:** 2020-08-24

**Authors:** J. Wang, S. Sun, Y. Chen, D. Chen, L. Sang, X. Xie

**Affiliations:** Institute of Animal Husbandry and Veterinary Medicine, Fujian Academy of Agricultural Sciences, Fuzhou, Fujian, People's Republic of China

**Keywords:** Antimicrobial susceptibility, *Bordetella bronchiseptica*, rabbit, multi-locus sequence typing, virulence genes

## Abstract

*Bordetella bronchiseptica* is a potential zoonotic pathogen, which mainly causes respiratory diseases in humans and a variety of animal species. *B*. *bronchiseptica* is one of the important pathogens isolated from rabbits in Fujian Province. However, the knowledge of the epidemiology and characteristics of the *B. bronchiseptica* in rabbits in Fujian Province is largely unknown. In this study, 219 *B*. *bronchiseptica* isolates recovered from lung samples of dead rabbits with respiratory diseases in Fujian Province were characterised by multi-locus sequencing typing, screening virulence genes and testing antimicrobial susceptibility. The results showed that the 219 isolates were typed into 11 sequence types (STs) including five known STs (ST6, ST10, ST12, ST14 and ST33) and six new STs (ST88, ST89, ST90, ST91, ST92 and ST93) and the ST33 (30.14%, 66/219), ST14 (26.94%, 59/219) and ST12 (16.44%, 36/219) were the three most prevalent STs. Surprisingly, all the 219 isolates carried the five virulence genes (*fhaB*, *prn*, *cyaA*, *dnt* and *bteA*) in the polymerase chain reaction screening. Moreover, the isolates were resistant to cefixime, ceftizoxime, cefatriaxone and ampicillin at rates of 33.33%, 31.05%, 11.87% and 3.20%, respectively. This study showed the genetic diversity of *B*. *bronchiseptica* in rabbits in Fujian Province, and the colonisation of the human-associated ST12 strain in rabbits in Fujian Province. The results might be useful for monitoring the epidemic strains, developing preventive methods and preventing the transmission of epidemic strains from rabbits to humans.

## Introduction

*Bordetella bronchiseptica* can infect humans and a number of animal species [1]. The infection of *B. bronchiseptica* in immunocompetent humans is rare. However, the pathogen was reported to associate with respiratory diseases in immunosuppressed humans [2–4]. In animals, the pathogen is also associated with respiratory diseases, such as canine infectious respiratory disease syndrome in dogs, tracheobronchitis in cats, atrophic rhinitis in pigs, pneumonia in horses and snuffles in rabbits [5–9]. It has been showed that humans could be infected with *B. bronchiseptica* by direct contact with infected animals (swine, dog, rabbit and cat), causing diseases including whooping cough, bronchitis, bronchopneumonia, pneumonia and peritonitis [10–12]. Therefore, more efforts should be endeavoured to understand the epidemiology and characteristics of *B. bronchiseptica* in animals, which will help in preventing the transmission of *B. bronchiseptica* from animals to humans.

Fujian Province, in the southeast of China, is one of the important rabbit-farming areas in China [13]. The number of live rabbits farmed was about 9.4 million by the end of 2018 (Fujian Statistical Yearbook, 2019). In Fujian Province, all the rabbits are used for food, and the rabbit meat production reached 14.1 thousand tonnes in 2018 (Fujian Statistical Yearbook, 2019). Traditionally, the hair and the abdominal organs (except for heart and liver) of the rabbit are removed and the slaughter rate is around 75%.

*B. bronchiseptica* spreads rapidly in rabbits and the infection is often persistent [14]. To the best of our knowledge, *B. bronchiseptica* is a common pathogen isolated from rabbits with respiratory diseases in Fujian Province. However, information on the prevalence and characteristics of *B*. *bronchiseptica* in rabbits in Fujian Province is limited. In this study, *B. bronchiseptica* strains were recovered from the lung samples of dead rabbits with respiratory diseases. The isolates were characterised by multi-locus sequencing typing, screening the virulence genes and testing the antimicrobial susceptibility.

## Methods

### Ethics statement

Lung samples were collected from dead rabbits with respiratory diseases in accordance with the guidelines issued by the Research Ethics Committee of the Institute of Animal Husbandry and Veterinary Medicine, Fujian Academy of Agriculture Sciences (FAAS). This study was also approved by the Research Ethics Committee of the Institute of Animal Husbandry and Veterinary Medicine, and the approved number is FAAS-AHVM2017-0511.

### Sample collection and *B. bronchiseptica* isolation

Nine rabbit farms in Fuzhou, Longyan and Nanping cities in Fujian Province were included for sampling. The two rabbit farms in Fuzhou raise Fujian Yellow rabbit (local rabbit breed), the three rabbit farms in Longyan raise Fujian White rabbit (local rabbit breed), the other two rabbit farms in Longyan raise Minxinan Black rabbit (local rabbit breed) and the two rabbit farms in Nanping raise Hyplus rabbit (foreign rabbit breed). The whole lungs were collected from dead rabbits with respiratory diseases. In all, 833 whole lungs were collected from August 2017 to December 2019.

In order to isolate *B*. *bronchiseptica*, 1 g lung tissue (lower lobe) from each whole lung was mixed with 1 ml of sterile phosphate buffer saline and homogenised to make 50% suspension. One hundred microlitres of suspension was spread on brain heart infusion (BHI) agar plate (containing 1% glycerol and 10% defibrinated sheep blood) and incubated for 48–72 h at 37 °C. The suspected smooth, round, greyish white and shiny colonies with the diameter around 1 mm were picked up and purified. Five isolates of Gram negative, oxidase and ornithine decarboxylase positives, and glucose fermentation negative were further confirmed by sequencing of the *16S rRNA* genes. One isolate from each lung sample was selected as representative for further characterisation.

### Multi-locus sequence typing

The isolates were analysed by multi-locus sequence typing (MLST) as described in PubMLST website (https://pubmlst.org/). Briefly, seven house-keeping genes including *adk*, *fumC*, *glyA*, *tyrB*, *icd*, *pepA* and *pgm* were amplified and sequenced. The seven allelic numbers were given by comparing the seven house-keeping genes of the isolate to the corresponding genes in the PubMLST database. The sequence types (STs) of the isolates were defined according to the seven allelic numbers.

### Virulence gene detection

Five well-characterised virulence genes that thought to be involved in interactions with the host were screened in the isolates, including filamentous haemagglutinin (FHA) (*fhaB*), pertactin (*prn*), adenylate cyclase-haemolysin toxin (*cyaA*), dermonecrotic toxin (*dnt*) and the *Bordetella* type-III secretion system effector A (*bteA*) [15–19]. The primers for amplification of the five virulence genes were designed based on the corresponding genes in the NCBI database (https://www.ncbi.nlm.nih.gov/) (Table 1). Polymerase chain reaction (PCR) mixtures comprised of 25 μl 2× *EasyTaq* PCR SuperMix (TransGen Biotech, Beijing, China), 0.2 μM of each forward and reverse primer and 100 ng bacterial genome DNA in a final volume of 50 μl. The cycling conditions for PCR assays were 94 °C for 5 min and 35 cycles of 94 °C for 30 s, 59 °C for 30 s and 72 °C for 30 s, followed by a final elongation step of 72 °C for 5 min. The *B*. *bronchiseptica* strain MH001 and the sterile ddH_2_O were included as the positive and negative controls, respectively [20]. The PCR products were sequenced to confirm the identities.

**Table 1.** Primers used for amplification of the five virulence genes in the *B*. *bronchiseptica* isolates

Genes	Descriptions	Primer sequence (5′–3′)	Product size (bp)	Accession numbers of reference genes
*fhaB*	Filamentous haemagglutinin	F: gcgcagaacatcaccaatg	475	CP024173 (range from 3 041 205 to 3 042 932)
		R: tgaaatactccatggcggac		
*prn*	Pertactin	F: gacctcgctcagtcgatc	555	AJ245927
		R: gaagacattcatgcggaacag		
*cyaA*	Adenylate cyclase-haemolysin toxin	F: ctacgagcagttcgagtttc	377	Z37112
		R: tattcatgtcgccgtcgta		
*dnt*	Dermonecrotic toxin	F: tgatcctgcagtggttgatc	491	U59687
		R: atcggcatacgccagatc		
*bteA*	*Bordetella* type-III secretion system effector A	F: tgttgagcaacaacgtcaatc	474	HE974463
		R: tatgcaggtcttcgaggttc		

### Antimicrobial susceptibility test

The antimicrobial susceptibility test of the isolates were conducted by using disc diffusion method on BHI agar plate (containing 1% glycerol and 10% defibrinated sheep blood) according to the Clinical and Laboratory Standards Institute (CLSI) standards. Ten antibiotics with the stated concentrations (μg/disc) were included: ampicillin (10), cefixime (5), cefotaxime (30), ceftizoxime (30), gentamycin (10), kanamycin (30), streptomycin (10), ofloxacin (5), ciprofloxacin (5) and norfloxacin (10). The results were interpreted based on the breakpoints for *Enterobacteriaceae* of the CLSI standards [21, 22]. The *Staphylococcus aureus* ATCC 29213 was included as the quality control.

## Results

### *B. bronchiseptica* isolation and identification

Two hundred and nineteen *B. bronchiseptica* isolates were recovered from the 833 whole lung samples of dead rabbits with respiratory diseases. The *B. bronchiseptica* isolates could be recovered from the all nine rabbit farms and the all four rabbit breeds (Table 2). The infection rates of *B. bronchiseptica* in the nine rabbit farms and the four rabbit breeds ranged from 13.79% to 38.42% and 15.66% to 50.24%, respectively.

**Table 2.** Distribution, origin and STs of the 219 *B. bronchiseptica* isolates

Cities	Rabbit farms	Rabbit breeds	No. of samples	No. of isolates	ST:No. of isolates
Fuzhou	Farm A	Fujian yellow rabbit	177	68	ST12:19; ST14:34
					ST33:12; ***ST89*:2**
					***ST93*:1**
	Farm B	Fujian yellow rabbit	113	37	ST6:12; ST14:8
					ST33:15; ***ST90*:2**
Longyan	Farm C	Fujian white rabbit	91	22	ST6:8; ST10:7
					ST33:6; ***ST88*:1**
	Farm D	Fujian white rabbit	102	21	ST12:14; ST14:6
					***ST91*:1**
	Farm E	Fujian white rabbit	68	15	ST10:6; ST33:9
	Farm F	Minxinan black rabbit	75	18	ST14:3; ST33:13
					***ST90*:2**
	Farm G	Minxinan black rabbit	79	18	ST6:7; ST33:10
					***ST92*:1**
Nanping	Farm H	Hyplus rabbit	87	12	ST12:3; ST14:8
					ST33:1
	Farm I	Hyplus rabbit	41	8	ST6:6; ST10:2
Total			833	219	

The bold-italic characters indicate the new STs.

### Multi-locus sequence typing

**Fig. 1.**
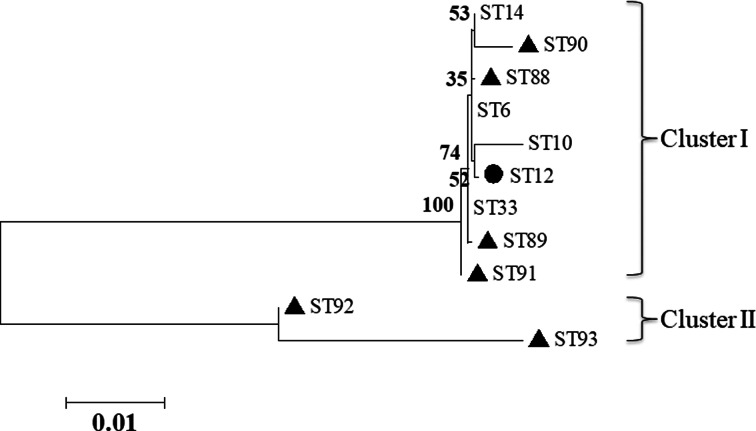
Phylogenetic tree of the 11 STs of *B. bronchiseptica* based on the concatenated seven house-keeping genes. The black filled triangles indicate the new STs. The black filled circle indicates the *B. bronchiseptica* strain of ST12 that can be isolated from both rabbits and humans.

The results of MLST analyses showed that the 219 *B. bronchiseptica* isolates were typed into 11 STs, with the ST33 (30.14%, 66/219), ST14 (26.94%, 59/219) and ST12 (16.44%, 36/219) were the three most predominant STs. Among the 11 STs, six new STs were detected including ST88, ST89, ST90, ST91, ST92 and ST93 (Table 2), and the MLST profiles of the six new STs had been submitted to the PubMLST database (https://pubmlst.org/bigsdb?db=pubmlst_bordetella_seqdef&page=profiles&scheme_id=3). The seven house-keeping genes of each of the isolate of the 11 STs were concatenated for phylogenetic analysis. A maximum likelihood tree was constructed (no. of bootstrap replications was 1000) by using the MEGA 5.0 software. The 11 STs were mainly grouped into two clusters, cluster I and cluster II. Cluster I contained nine STs including ST6, ST10, ST12, ST14, ST33, ST88, ST89, ST90 and ST91, whereas cluster II only contained two STs including ST92 and ST93 (Fig. 1).

### Virulence gene detection

Five virulence genes were screened in the 219 *B. bronchiseptica* isolates. The results showed that all the five virulence genes of *fhaB*, *prn*, *cyaA*, *dnt* and *bteA* were positive in the all 219 isolates.

### Antimicrobial susceptibility test

The results of antimicrobial susceptibility test showed that most of the *B. bronchiseptica* isolates in this study were susceptible or intermediate susceptible to streptomycin, ciprofloxacin, gentamycin, ofloxacin, kanamycin and norfloxacin, and the sensitive rates to the six kinds of drugs were 100%, 99.09%, 98.63%, 96.80%, 96.34% and 21%, respectively (Table 3). The isolates resistant to cefixime, ceftizoxime, cefatriaxone and ampicillin were observed, and the resistance rates to these four kinds of drugs were 33.33%, 31.05%, 11.87% and 3.20%, respectively (Table 3). No isolate of multi-drug resistant was detected.

**Table 3.** Antimicrobial susceptibility test of the 219 *B. bronchiseptica* isolates

Antibiotics	No. of isolates			Percentage of resistance or sensitive	
	R	I	S	Resistance rate	Sensitive rate
Ampicillin	7	210	2	3.20	0.91
Cefixime	73	145	1	33.33	0.46
Cefatriaxone	26	185	8	11.87	3.65
Ceftizoxime	68	151	0	31.05	0
Gentamycin	0	3	216	0	98.63
Kanamycin	0	8	211	0	96.34
Streptomycin	0	0	219	0	100
Ofloxacin	0	7	212	0	96.80
Ciprofloxacin	0	2	217	0	99.09
Norfloxacin	0	173	46	0	21

‘R’ represents resistance; ‘I’ represents intermediate; ‘S’ represents susceptible.

## Discussion

The present study described the epidemiology and characteristics of *B. bronchiseptica* in rabbits in Fujian Province. The results showed that *B. bronchiseptica* was prevalent in the nine rabbit farms and the four rabbit breeds, and the infection rate of *B. bronchiseptica* in rabbits in Fujian Province was as high as 26.29% (219/833). The results suggested that *B. bronchiseptica* is an important pathogen causing respiratory diseases in rabbits in Fujian Province.

The 219 *B. bronchiseptica* isolates in this study were typed into five known STs (ST6, ST10, ST12, ST14 and ST33) and six new STs (ST88, ST89, ST90, ST91, ST92 and ST93). In the PubMLST database (https://pubmlst.org/bordetella/), *B. bronchiseptica* strains of ST6 are isolated from rabbits in Switzerland, strains of ST10 are isolated from rabbits in USA, strains of ST12 are isolated from rabbits in USA and Denmark, and strains of ST14 are isolated from rabbits in USA and UK. However, strains belonging to ST33 that the most prevalent ST (30.14%, 66/219) in rabbits in Fujian Province are isolated from seal, dog, leopard and horse, but not from rabbits. Notably, strains of ST12 could also be isolated from humans, suggesting the potential zoonotic transmission between rabbits and humans [11]. Moreover, the five known STs (ST6, ST10, ST12, ST14 and ST33) are grouped into the clonal complex of ST6 complex (the ST6 is the putative founder) in the PubMLST database. By comparing the MLST profiles of the six new STs (ST88, ST89, ST90, ST91, ST92 and ST93) with that of ST6, ST88 showed the most genetic closely to ST6 (ST88 is the single locus variant of ST6) [23].

Expression of the virulence factors facilitates the invasion of *B. bronchiseptica* in hosts [24]. Surprisingly, all the 219 *B. bronchiseptica* isolates in this study carried the five screened virulence genes of *fhaB*, *prn*, *cyaA*, *dnt* and *bteA*. The *fhaB* and *prn* genes encode the FHA and pertactin (PRN), respectively. The FHA and PRN are important adhesins expressed in the genetic closely related species of *B. pertussis*, *B. parapertussis* and *B. bronchiseptica*, which mediates the bacterial adhesion of host cells [25, 26]. It should be aware that the PRN-deficient *B. pertussis* had emerged, and the losses of the *prn* gene expression in the strains might be driven by using of acellular vaccine containing PRN [27]. The *cyaA* gene encodes the multifunctional adenylate cyclase-haemolysin (AC-Hly) toxin, which possess adenylate cyclase activity, pore-forming activity and cell invasive activity [17]. It was showed that the AC-Hly toxin expressed in the *B. bronchiseptica* strains isolated from human and rabbit was responsible for the lethality of the intranasally infected mice [11]. The *dnt* gene encodes the dermonecrotic toxin (DNT), and the toxin is a well-recognised causative factor inducing atrophic rhinitis and bronchopneumonia in pigs [18]. Interestingly, the ability to express DNT is varied among strains. It was showed that the larger amount of DNT was produced in pig isolates than in a rabbit isolate RB50 [28]. The *bteA*, also known as *bopC*, encodes the *Bordetella* type III secretion system effector A (BteA), which is an important effector secreted from the type III secretion system [19, 29]. It was showed that BteA could induce the necrotic cell death and inhibit the macrophage phagocytosis [19, 29]. Taking together, the 219 *B. bronchiseptica* isolates carrying the five virulence genes in this study were the potential pathogen causing severe respiratory infection in rabbits.

Antibiotics play an important role in prevention and treatment of infections caused by *B. bronchiseptica* [30–32]. However, the widespread use of antibiotics for preventing and treating the bacterial infections leads to the emergence of antibiotic-resistant strains [32–34]. It was demonstrated that *B. bronchiseptica* strains isolated from swine in Germany were resistant to ampicillin, and *B. bronchiseptica* strains isolated from companion animals in Germany and other European countries showed decreased susceptibility to *β*-lactam antibiotics and cephalosporins [35]. In consistence with this study, *B. bronchiseptica* isolates showing resistant to ampicillin and cephalosporins were also observed in this study. It was showed that the production of beta-lactamases and the reduced membrane permeability to cephalosporins might result in the resistance to ampicillin and cephalosporins, respectively [36]. Therefore, it should be aware that the widespread use of antibiotics to control the infection caused by *B. bronchiseptica* in rabbits is unsustainable.

The characteristics of the *B. bronchiseptica* strains isolated from dead rabbits with respiratory disease in Fujian Province were described in this study. The results showed that *B. bronchiseptica* was an important pathogen causing respiratory diseases in rabbits in Fujian Province, and that *B. bronchiseptica* stain of ST12 that can infect humans was also isolated from rabbits in Fujian Province. Unexpectedly, *B. bronchiseptica* isolates belonging to six new STs (ST88, ST89, ST90, ST91, ST92 and ST93) were detected, and *B. bronchiseptica* isolates resistance to cefixime, ceftizoxime, cefatriaxone and ampicillin were also detected. The results might play important roles in tracking the epidemic strains in rabbits, controlling the *B. bronchiseptica* infections in rabbits and preventing the potential rabbit–human transmission events.

## Data Availability

Requests for access to the data that support this study should be made to the corresponding author, XX.
